# Hospitalisations for Pelvic Inflammatory Disease Temporally Related to a Diagnosis of Chlamydia or Gonorrhoea: A Retrospective Cohort Study

**DOI:** 10.1371/journal.pone.0094361

**Published:** 2014-04-17

**Authors:** Joanne Reekie, Basil Donovan, Rebecca Guy, Jane S. Hocking, Louisa Jorm, John M. Kaldor, Donna B. Mak, David Preen, Sallie Pearson, Christine L. Roberts, Louise Stewart, Handan Wand, James Ward, Bette Liu

**Affiliations:** 1 The Kirby Institute, UNSW Australia, Sydney, Australia; 2 Sydney Sexual Health Centre, Sydney Hospital, Sydney, Australia; 3 Melbourne School of Population and Global Health, University of Melbourne, Melbourne, Australia; 4 Centre for Health Research, University of Western Sydney, Sydney, Australia; 5 School of Medicine, University of Notre Dame, Fremantle, Australia; 6 Centre for Health Services and Research, University of Western Australia, Crawley, Australia; 7 Faculty of Pharmacy and School of Public Health, University of Sydney, Sydney, Australia; 8 Kolling Institute of Medical Research, University of Sydney, Sydney, Australia; 9 Centre for Population Health Research, Curtin University, Perth, Australia; 10 Baker IDI, Alice Springs, Northern Territory, Australia; 11 School of Public Health and Community Medicine, UNSW Australia, Sydney, Australia; 12 The Sax Institute, Sydney, Australia; Columbia University, United States of America

## Abstract

**Objectives:**

The presence and severity of pelvic inflammatory disease (PID) symptoms are thought to vary by microbiological etiology but there is limited empirical evidence. We sought to estimate and compare the rates of hospitalisation for PID temporally related to diagnoses of gonorrhoea and chlamydia.

**Methods:**

All women, aged 15–45 years in the Australian state of New South Wales (NSW), with a diagnosis of chlamydia or gonorrhoea between 01/07/2000 and 31/12/2008 were followed by record linkage for up to one year after their chlamydia or gonorrhoea diagnosis for hospitalisations for PID. Standardised incidence ratios compared the incidence of PID hospitalisations to the age-equivalent NSW population.

**Results:**

A total of 38,193 women had a chlamydia diagnosis, of which 483 were hospitalised for PID; incidence rate (IR) 13.9 per 1000 person-years of follow-up (PYFU) (95%CI 12.6–15.1). In contrast, 1015 had a gonorrhoea diagnosis, of which 45 were hospitalised for PID (IR 50.8 per 1000 PYFU, 95%CI 36.0–65.6). The annual incidence of PID hospitalisation temporally related to a chlamydia or gonorrhoea diagnosis was 27.0 (95%CI 24.4–29.8) and 96.6 (95%CI 64.7–138.8) times greater, respectively, than the age-equivalent NSW female population. Younger age, socio-economic disadvantage, having a diagnosis prior to 2005 and having a prior birth were also associated with being hospitalised for PID.

**Conclusions:**

Chlamydia and gonorrhoea are both associated with large increases in the risk of PID hospitalisation. Our data suggest the risk of PID hospitalisation is much higher for gonorrhoea than chlamydia; however, further research is needed to confirm this finding.

## Introduction

Pelvic inflammatory disease (PID) is a common condition in women of reproductive age and can lead to infertility, ectopic pregnancy, recurrent PID, and chronic pelvic pain[1]–[3]. Although PID can be caused by multiple microorganisms, in young women PID commonly results from the ascent of sexually transmitted *Chlamydia trachomatis* (chlamydia) or *Neisseria gonorrhoea* (gonorrhoea) infections from the cervix to the upper genital tract[4]–[6].

Chlamydia is the most frequently reported notifiable sexually transmitted infection (STI), in Australia and a number of other countries, with rates of diagnoses increasing consistently over the past decade[7]–[9]. Over this same period the rates of gonorrhoea diagnoses have been more variable[7]–[9]. Although gonorrhoea is the second most common notifiable STI in many countries, rates are substantially lower than those of chlamydia[8], [10].

As the presence and severity of PID symptoms are thought to vary depending on the causative organism, but there is little published empirical evidence[11], this study aimed to estimate and compare the incidence of PID hospitalisation temporally related to diagnoses of chlamydia and gonorrhoea in a population-based sample of women of reproductive age in the state of New South Wales (NSW), Australia.

## Methods

We used a retrospective cohort design, to measure the incidence of hospitalised PID associated with a reported diagnosis of gonorrhoea or chlamydia. The study was approved by the NSW Population and Health Services Research Ethics Committee and the University of New South Wales Human Research Ethics Committee. All participant records/information was anonymized and de-identified prior to analysis.

### Data

This study was conducted in the Australian state of New South Wales (NSW): (total population 7.23 million in 2010) by probabilistically linking data, using personal identifying details, from four registries; the Notifiable Conditions Information Management System (NCIMS), the Admitted Patient Data Collection (APDC), the Perinatal Data Collection (PDC), and the Registry of Births, Deaths, and Marriages (RBDM). The linkage was conducted by the NSW Centre for Health Record Linkage (CHeReL), independent of the study investigators, and the de-identified but linkable data, were made available to the investigators for analysis (http://www.cherel.org.au/how-record-linkage-works).

The NCIMS is a statutory whole of population surveillance system, which records details of medical conditions notifiable under the Public Health Act 1991, including all laboratory diagnoses of gonorrhoea from 1994 and genital chlamydia from 1998 onwards in NSW. Records for each case include the estimated onset date, specimen collection date, and notification date. The APDC records all admitted patient services provided by New South Wales Public Hospitals, Public Psychiatric Hospitals, Public Multi-Purpose Services, Private Hospitals, and Private Day Procedures Centre in NSW. Each record includes the principal diagnosis and up to 49 additional diagnoses coded according to the International Classification of Diseases Australian Modification version 10 (ICD10-AM) [12] and admission and discharge dates. Records were available for linkage from July 2000. The PDC contains linkable records for each birth in NSW of at least 400 grams birth weight, or at least 20 weeks gestation, including details about the pregnancy and the date of birth from 1994. The RBDM contains a record of all deaths in NSW and the date of death. Data up to 31st December 2008 were extracted from each register.

### Analysis

The cohort for analyses included all women resident in NSW, aged between 15 and 45 years from the NCIMS records who had a diagnosis of chlamydia or gonorrhoea on or after 01/07/2000. The date of chlamydia or gonorrhoea diagnosis was calculated as the earliest of the reported onset date, specimen collection date, or notification date. Women were classified as having had *chlamydia only*, if they had a diagnosis of chlamydia and no prior notification of gonorrhoea, *gonorrhoea only*, if they had a diagnosis of gonorrhoea and no prior notification for chlamydia, or *both*, if they had a diagnosis of either chlamydia or gonorrhoea and had a previous/concurrent notification for the other. The primary outcome of hospitalisation for PID was determined for each woman based on their linked APDC records. Hospitalisation for PID was defined as a linked APDC record where the primary ICD10-AM diagnosis code was N70, N71, N73, N74.3, N74.4, A54.0, A54.1, A54.2, A56.0, A56.1, or A56.2 (see Text S1 for ICD-10 definitions).

**Figure 1:**
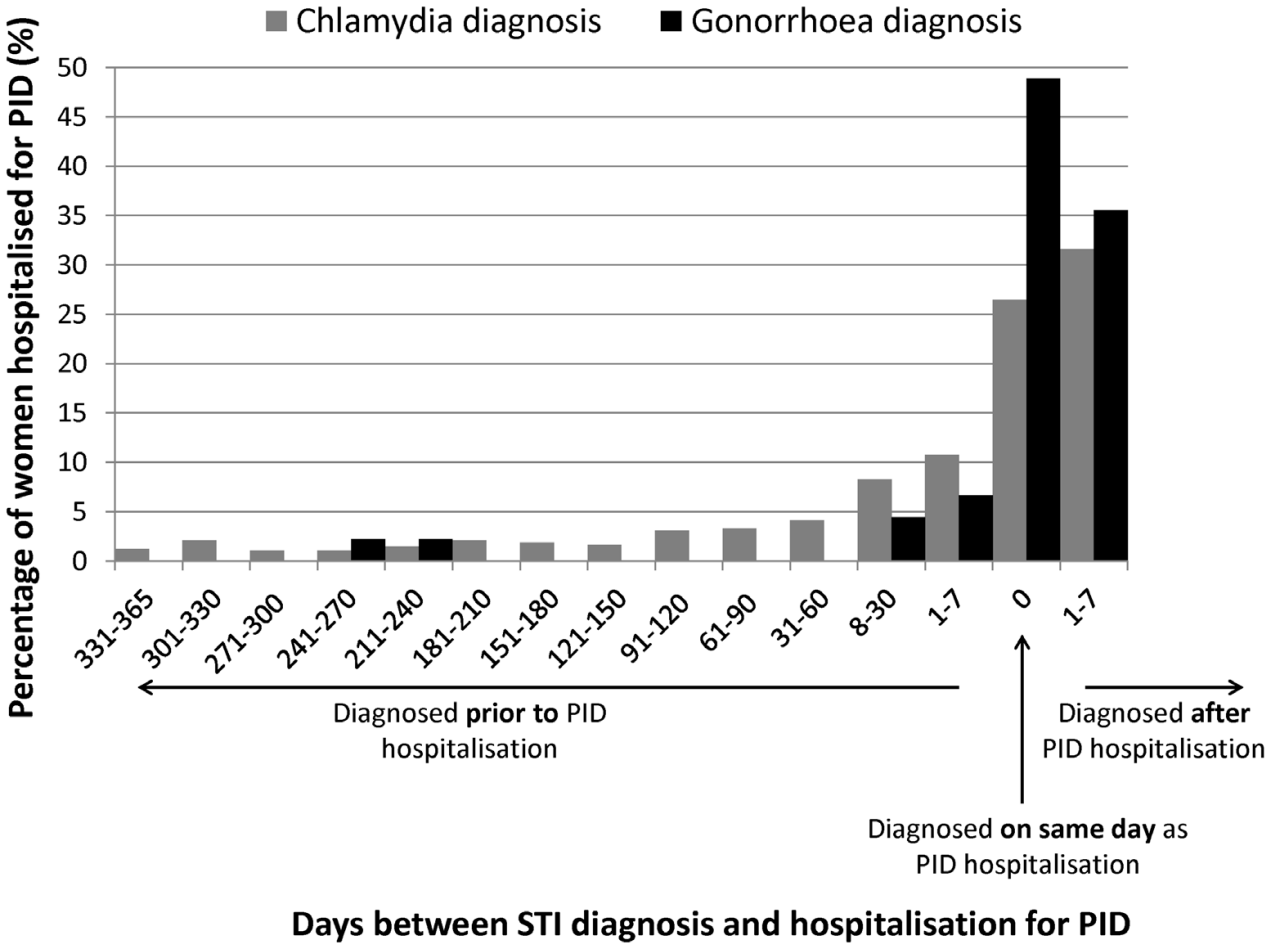
Distribution of time between chlamydia or gonorrhoea diagnosis and admission to hospital for pelvic inflammatory disease (PID).

Among the women who were hospitalised for PID, we examined the time between diagnosis of chlamydia or gonorrhoea and hospital admission date. A large number of the chlamydia and gonorrhoea diagnoses were recorded in the week following hospital admission for PID (see Figure 1). Therefore, where the date of diagnosis occurred on the admission date or within the 7 days after admission, this was recoded as occurring the day before hospitalisation as it was assumed that the diagnosis of PID was related to the chlamydia or gonorrhoea diagnosis. Information for 58% (n = 281) of women with chlamydia who were hospitalised for PID (0.74% of all women with a chlamydia diagnosis) and 84% (n = 38) of women with gonorrhoea who were hospitalised for PID (3.7% of all women with a gonorrhoea diagnosis) were recoded in this way.

The incidence of PID in the 12 months after the chlamydia or gonorrhoea diagnosis was calculated per 1000 person-years of follow-up (PYFU). Follow-up time was calculated from the date of diagnosis and censored at either the date of hospitalisation for PID, one year after the diagnosis, death, or the last date there were records (31/12/2008), whichever occurred first. Women recorded with a diagnosis of chlamydia or gonorrhoea who then went on to receive a diagnosis for the other infection during their 12 months of follow-up, were able to contribute follow-up initially in the category of the first, single diagnosis and then in the “both” category after the second diagnosis.

We compared the incidence of PID hospitalisations among women with a diagnosis of chlamydia, gonorrhoea or both to PID hospitalisations in the general population, using standardised incidence ratios (SIR). Age specific PID hospitalisation rates for the NSW female population were calculated for NSW using census population denominators[13]. SIRs were standardised using rates specific for age group (15–19, 20–24, and 25–30 years) and single calendar year (2000–2008).

Finally Poisson regression analysis was used to identify factors independently associated with hospitalisation for PID among women with a diagnosis of chlamydia or gonorrhoea. Factors considered included age at diagnosis, year of diagnosis (<2005, ≥2005), socioeconomic group (in tertiles based on a standard Australian census classification[14]), geographic classification of area of residence (major city, inner regional, outer regional/remote)[15]) and having given birth in NSW prior to the STI diagnosis (using linked records in the PDC).

We then assessed whether the associations were consistent for women diagnosed prior to 2005 versus women diagnosed in 2005 onwards as there have been changes in testing guidelines and the management of PID over this period[4]; in women aged ≤25 years versus women aged >25 years, as effects may differ with age; in women resident in major cities vs. women not resident in a major city, as access/proximity to a hospital may affect a women's likelihood of being hospitalised for PID; and in parous versus nulliparous women. A sensitivity analysis was also conducted excluding all women whose date of chlamydia or gonorrhoea diagnosis had been recoded in the main analysis, and any women who had a hospitalisation for PID prior to their STI diagnosis.

## Results

A total of 39,254 women had a record of at least one chlamydia or gonorrhoea diagnosis between 01/07/2000 and 31/12/2008. The majority (97.3%) of these had a diagnosis of chlamydia with no prior gonorrhoea diagnosis (Table 1), 2.6% had a diagnosis of gonorrhoea with no prior chlamydia diagnoses, and 0.1% had had a diagnosis of either chlamydia or gonorrhoea with a notification prior to 01/07/2000 for the other. Most diagnoses were made during the latter half of the observation period (61% after 01/01/2005).

**Table 1:** Characteristics of women aged 15–45 at the time of chlamydia or gonorrhoea diagnosis, New South Wales 2000–2008.

		Chlamydia only		Gonorrhoea only		Both	
Total (N, % of total)		38193	97.3	1015	2.6	46	0.1
Age (Median, IQR)	Years	22.4	19.5–26.8	26.1	21.2–37.8	23	20.4–37.9
Socioeconomic disadvantage* (N, %)	Least disadvantaged	12096	31.7	439	43.3	9	19.6
	Middle tertile	12629	33.1	313	30.8	12	26.1
	Most disadvantaged	13119	34.4	254	25.0	24	52.2
	Unknown	349	0.9	9	0.9	1	2.2
Area of residence+ (N,%)	Major city	26908	70.5	826	81.4	23	50.0
	Inner regional	8779	23.0	136	13.4	14	30.4
	Outer regional/remote	2170	5.7	43	4.2	8	17.4
	Unknown	336	0.9	10	1.0	1	2.2
Given birth prior to diagnosis (N, %)		5027	13.2	175	17.2	28	60.9
Prior hospitalisation for PID (N, %)		159	0.4	4	0.4	1	2.2
Prior hospitalisation for ectopic pregnancy (N, %)		102	0.3	3	0.3	0	0.0
Prior hospitalisation for miscarriage (N,%)		336	0.9	14	1.4	1	2.2
Prior hospitalisation for infertility treatment (N, %)		93	0.2	2	0.2	0	0.0

*based on SEIFA score (see methods),

+ based on ARIA score (see methods), IQR: Interquartile range.

As shown in table 1, women with a chlamydia diagnosis were significantly younger than those with gonorrhoea (median age 22 vs. 26 years, p<.0001). They were also more likely to live in a socio-economically disadvantaged area (34% vs. 25%, p<.0001) and less likely to live in a major city (71% with chlamydia and 81% gonorrhoea, p<.0001). Fewer women with chlamydia had given birth prior to their STI diagnosis than those with gonorrhoea (13% vs. 17%, p<.0001) perhaps due to their younger age. A small percentage of women had a prior hospitalisation record for PID (0.4% vs. 0.4%, p = 0.91), ectopic pregnancy (0.3% vs. 0.3%, p = 0.75), miscarriage (0.9% vs. 1.4%, p = 0.09), or infertility (0.2% vs. 02%, p = 0.76) and these proportions were similar among women diagnosed with either chlamydia or gonorrhoea.

The 38,193 women with a chlamydia diagnosis at baseline were followed for a total of 34,833 PYFU. Over this time 1567 (4.1%) had at least one repeat diagnosis of chlamydia, 48 (0.1%) had a subsequent diagnosis of gonorrhoea and 483 were hospitalised for PID, giving a PID incidence rate (IR) of 13.9 per 1000 PYFU (95%CI 12.6–15.1). Among the 1015 women with a gonorrhoea diagnosis, during 886 PYFU, 10 (1.0%) had at least one repeat diagnosis of gonorrhoea, 31 (3.1%) had a subsequent diagnosis of chlamydia and 45 were hospitalised for PID; IR 50.8 per 1000 PYFU (95%CI 36.0–65.6).

**Figure 2:**
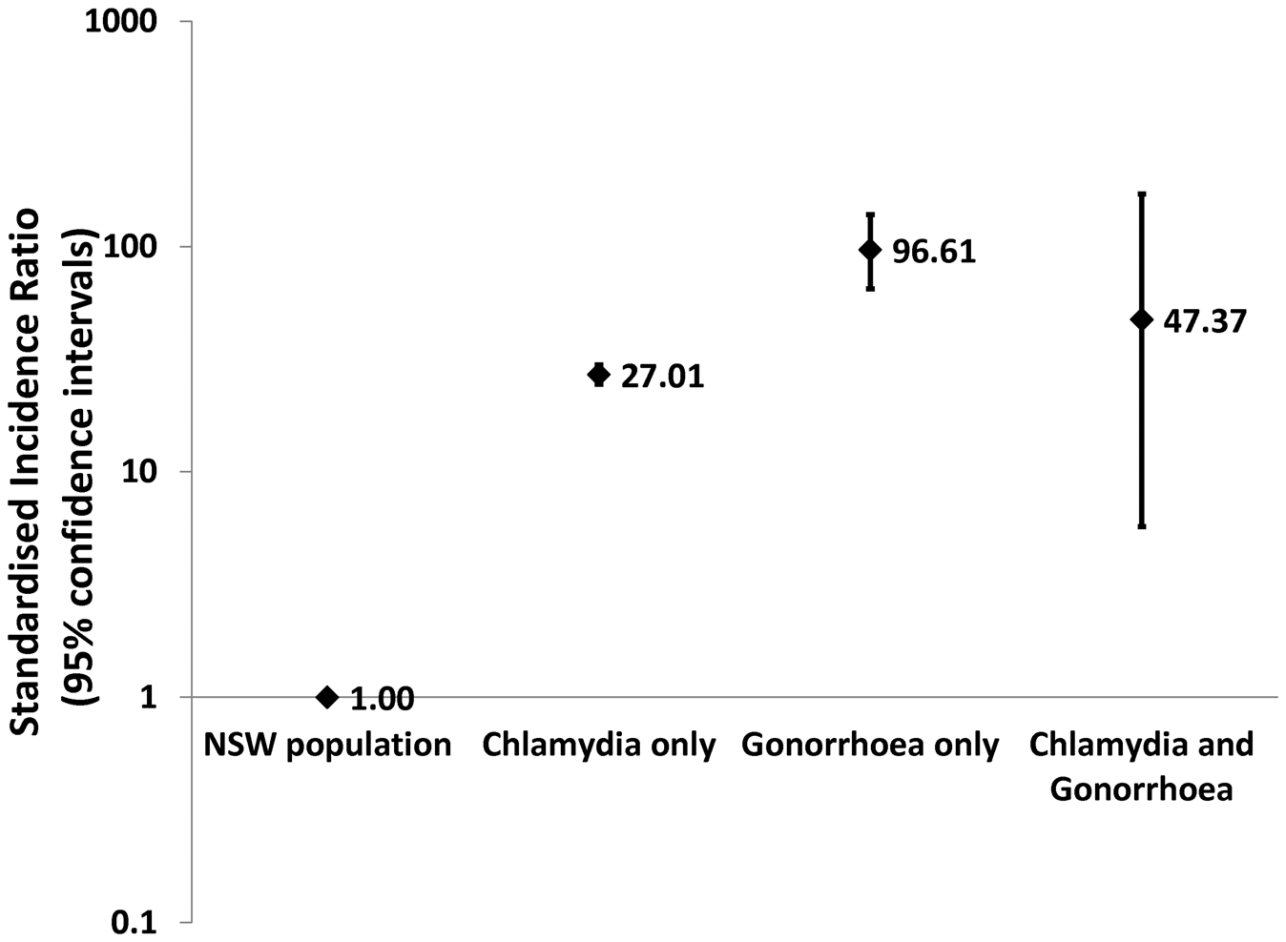
Standardised* incidence ratio (SIR) for hospitalisation for pelvic inflammatory disease (PID) in women in New South Wales (NSW) aged 15–30. *standardised for age and year of follow-up.

Figure 2 shows the SIR for hospitalisation for PID in women with a diagnosis of chlamydia, gonorrhoea or both. Compared to the age and calendar year equivalent NSW female population, the annual incidence of PID hospitalisation was 27.0 (95%CI 24.4–29.8) times greater among women with a chlamydia diagnosis and 96.6 (95%CI 64.7–138.8) times greater among women with a gonorrhoea diagnosis.

Table 2 shows the factors associated with hospitalisation for PID among the 39,254 women with a chlamydia and/or gonorrhoea diagnosis. After adjustment for age, year of diagnosis, socio-economic disadvantage, area of residence and previous birth, gonorrhoea remained a stronger risk factor than chlamydia for hospitalisation for PID (adjusted incidence rate ratio [IRR] 3.48, 95%CI 2.51–4.82, p<.0001). In addition, older age (IRR 0.77 per 10 years, 0.65–0.91, p = 0.002), being diagnosed from 2005 onwards (IRR 0.77, 95% CI 0.65–0.92, p = 0.003) and living in an inner regional area compared to a major city (IRR 0.72, 95%CI 0.57–0.91, p = 0.005) were associated with a lower rate of PID hospitalisation. Women from the most socio-economically disadvantaged areas compared to those from the least disadvantaged area (IRR 1.33, 95%CI 1.02–1.74, p = 0.03) and those who had previously given birth (IRR 2.71, 95%CI 2.18–3.36, p<.0001) had a higher rate of hospitalisation for PID.

**Table 2:** Factors associated with hospitalisation for PID.

		N		Univariate			Multivariate		
		PID	PYFU	IRR	95% CI	p-value	IRR	95% CI	p-value
Diagnosis	Chlamydia only	483	34833	1.00			1.00		
	Gonorrhoea only	45	886	3.66	2.68–5.00	<.0001	3.48	2.51–4.82	<.0001
	Both	2	96	1.50	0.37–6.08	0.57	1.05	0.25–4.33	0.94
Age, in years	15–19	177	10327						
	20–24	164	13366						
	25–30	93	6511						
	>30	96	5612						
	Per ten years older			0.96	0.83–1.12	0.65	0.77	0.65–0.91	0.002
Year of diagnosis	<2005	257	14999	1.00			1.00		
	≥2005	273	20817	0.86	0.82–0.89	<.0001	0.77	0.65–0.92	0.003
Socioeconomic disadvantage*	Least disadvantaged	131	11559	1.00			1.00		
	Middle tertile	188	11781	1.41	1.13–1.76	0.002	1.34	1.05–1.69	0.01
	Most disadvantaged	205	12142	1.49	1.20–1.86	0.0004	1.33	1.02–1.74	0.03
	Unknown	6	333	1.59	0.70–3.63	0.27			
Area of residence+	Major city	375	25320	1.00			1.00		
	Inner regional	105	8168	0.87	0.70–1.08	0.20	0.72	0.57–0.91	0.005
	Outer regional/remote	42	2009	1.41	1.02–1.95	0.03	1.08	0.76–1.55	0.65
	Unknown	8	319	1.69	0.83–3.44	0.14			
Prior birth	No	378	31117	1.00			1.00		
	Yes	152	4700	2.66	2.20–3.22	<.0001	2.71	2.18–3.36	<.0001

*based on SEIFA score (see methods),

+ based on ARIA score (see methods), individuals with unknown SEIFA or ARIA score were excluded from the multivariate analysis due to the high level of correlation.

The relative effects of gonorrhoea and chlamydia on PID incidence remained similar comparing analyses in parous versus nulliparous women, those resident in major cities versus not resident in major cities, in women aged ≤25 versus women aged >25, and women diagnosed prior to 2005 versus from 2005 onwards (results not shown). The tests for interactions between STI diagnosis and prior births, area of residence, age or year of diagnosis resulted in p-values of 0.96, 0.17, 0.22 and 0.05 respectively.

Sensitivity analysis excluding 472 (1.2%) women who had their STI notification date recoded or were hospitalised for PID prior to receiving an STI notification demonstrated that the difference in the rate of hospitalisation for PID between women with chlamydia and gonorrhoea was no longer significant (IRR 1.52, 95%CI 0.71–3.23, p = 0.28). There were 194 hospitalisations for PID during 34,706 PYFU in the year after a chlamydia notification and 7 hospitalisations for PID during 886 PYFU in the year after a gonorrhoea notification. Compared to the age and calendar year equivalent NSW female population, the annual incidence of PID hospitalisation was 10.9 (95%CI 9.3–12.7) and 13.4 (95%CI 3.6–34.2) times greater among women who had had a chlamydia or gonorrhoea diagnosis respectively.

## Discussion

This study, of over 39,000 women of reproductive age, found that the rates of hospitalisation for PID in the year after a diagnosis of chlamydia or gonorrhoea were substantially higher than in the general female population of the same age. Further, they suggest that the rate of PID hospitalisations related to a diagnosis of gonorrhoea is higher than that related to a chlamydia diagnosis. Younger age, socio-economic disadvantage, having a diagnosis prior to 2005 and having a prior birth were also associated with being hospitalised for PID in this population of women with a chlamydia or gonorrhoea diagnosis.

It is well documented that women with chlamydia infection are at an increased risk of a diagnosis of PID and hospitalisation for PID although estimates of the size of the risk differ substantially[16]–[19]. However, information on the incidence of PID hospitalisation after a diagnosis of gonorrhoea is comparatively sparse. Two smaller studies of respectively 307 sex workers and 99 incarcerated adolescents found that previous exposure to gonorrhoea was associated with an increased risk of PID[20], [21] with similar relative risks for PID following either a chlamydia or gonorrhoea diagnosis. Our study included all women in the state (with a population of over 7 million) with a chlamydia or gonorrhoea diagnosis and therefore is likely to provide a more representative comparison than many other reports.

We found women diagnosed in or after 2005 had a lower risk of hospitalisation compared to women diagnosed prior to this date. This finding is consistent with a recent Australian study and data from other countries showing that the number of PID hospitalisations among 15–29 year-old females has decreased over the last decade[22] and may have resulted from changes in the management of PID, with a move to increasing outpatient treatment[4], [19]. Younger age at chlamydia or gonorrhoea diagnosis was associated with an increased risk of PID in our study, which has been reported in other studies[23]. Living in a more socioeconomically disadvantaged area was also associated with an increased risk of being hospitalised for PID after a STI diagnosis. It has previously been reported that people from a more socioeconomically disadvantaged background are more likely to delay seeking treatment[24], and therefore be hospitalised for conditions that could have been avoided with the preventative care and early disease management delivered in a primary care setting[25]. This may explain the higher proportion of hospitalisations observed among women in our study with lower socioeconomic status. Women with a diagnosis of chlamydia or gonorrhoea who had previously given birth were also found to have an increased risk of hospitalisation for PID. We are not aware of other studies that have shown this association and it requires further investigation although an earlier study found that having had a prior birth was also associated with more repeat chlamydia infections[26]. We unfortunately lacked additional sexual risk behavioural data which may explain the association with prior births.

In this study population, the majority of hospitalisations for PID in women occurred close to the time of STI notification but this was particularly the case for gonorrhoea which implies a relatively short incubation period (see Figure 1). For chlamydia, a recent modelling study based on data from the POPI chlamydia screening trial suggested that PID may occur at a constant rate during the course of a chlamydia infection rather than immediately following infection[27]. It is therefore possible that the higher incidence of PID hospitalisation that we observed in women with gonorrhoea compared to chlamydia may be due to chlamydia having a more gradual onset of symptoms, therefore increasing the likelihood of antibiotics to have been administered and for hospitalisations to have been prevented. Although we did not have data on actual timing of infection and treatment to draw any firm conclusions a study in a Sydney Sexual Health clinic reported that 97% of women diagnosed with chlamydia received appropriate treatment[28].

Potential limitations include the fact that PID can be difficult to diagnose. Knowledge of a recent STI diagnosis may lead to bias towards a diagnosis of PID and some hospitalised cases have been shown to have been incorrectly classified[29]. However, as we used hospitalisation records recorded independent to the linked chlamydia and gonorrhoea diagnoses, any misclassification of PID diagnoses in these records, should not differ between women with a chlamydia or gonorrhoea diagnosis. Due to changes in testing practices, chlamydia diagnoses and the admission criteria for PID may have also changed over time[4], [10]. However when we adjusted our analysis for year of diagnosis and also examined the effects in subgroups our results were consistent.

We did not have information on testing for chlamydia and gonorrhoea but know that only a minority of women at risk of chlamydia or gonorrhoea are tested[30]. This would leave many women with these infections undiagnosed in the community and may result in an underestimate of the effect of both STIs on PID risk when making comparisons with population rates of PID. Additionally, there may be unmeasured or unknown confounders that could account for the differences observed between risk of hospitalisation for PID related to a diagnosis of chlamydia to that of gonorrhoea.

Finally in the sensitivity analysis, when we excluded women who had their diagnosis date recoded, we found that the rate of hospitalisation for PID was no longer significantly different for women with chlamydia and gonorrhoea. While the relatively small numbers of events in our sensitivity analyses make the findings difficult to interpret, it suggests that there may be the potential for the recoding to bias our results and further studies comparing rates of PID in women with chlamydia and gonorrhoea are needed to confirm the observations in our ‘main analysis’.

In Australia, chlamydia is at least ten times more commonly diagnosed than gonorrhoea[10] and therefore the potential for a relative increase in PID hospitalisations with gonorrhoea is far outweighed by the much greater absolute numbers of chlamydia diagnoses. However, our findings may have particular implications in populations such as the United States of America where gonorrhoea infections in both women and men are more common[8] and also for Aboriginal and Torres Strait Islander people resident in remote areas of Australia where prevalence's of chlamydia and gonorrhoea are roughly equal[31], [32].

In conclusion, hospitalisation rates for PID in women with either chlamydia or gonorrhoea (in either isolation or combination) were substantially higher than in the comparative NSW female population. Our results also suggest that PID hospitalisations are greater in women diagnosed with gonorrhoea than chlamydia, however further research is needed to confirm this observation and to better understand the mechanisms underlying the different rates.

## Supporting Information

Text S1 **ICD-10 Conditions.**
(DOCX)
