# Supplementary material for: Hospitalisations for Pelvic Inflammatory Disease Temporally Related to a Diagnosis of Chlamydia or Gonorrhoea: A Retrospective Cohort Study
Source: PLoS One. 2014 Apr 17;9(4):e94361. doi: 10.1371/journal.pone.0094361 (PMC3990571; doi:10.1371/journal.pone.0094361)
Supplement: Text S1 — ICD-10 Conditions. (DOCX) [file pone.0094361.s001.docx]

**Text S1 ICD-10 Conditions**

N70 (Acute salpingitis and oophoritis), N71 (Inflammatory disease of uterus, except cervix), N73 (Other female pelvic inflammatory diseases), N74.3 (Female gonococcal pelvic inflammatory disease), N74.4 (Female chlamydial pelvic inflammatory disease), A54.0 (Gonococcal infection of lower genitourinary tract without periurethral or accessory gland abscess), A54.1 (Gonococcal infection of lower genitourinary tract with periurethral and accessory gland abscess), A54.2 (Gonococcal pelviperitonitis and other gonococcal genitourinary infections), A56.0 (Chlamydial infection of lower genitourinary tract), A56.1 (Chlamydial infection of pelviperitoneum and other genitourinary organs), and A56.2 Chlamydial infection of genitourinary tract, unspecified).
